# Affections of the Testicle in Hereditary Syphilis

**Published:** 1893-02

**Authors:** George Carpenter

**Affiliations:** London


### AFFECTIONS OF THE TESTICLE IN HEREDITARY SYPHILIS.

---

BY GEORGE CARPENTER, LONDON.

(*The Practitioner, 1892, xlix., 187.*)

---

To sum up, then, it appears in the present state of our knowledge that

1. The testicles may be affected so slightly in congenital syphilis that it needs a microscope to detect the malady.

2. In a certain small percentage of cases the lesions are such that they can be detected by physical examination. These lesions may be present at birth, arise soon after, or make their appearance some months or years afterwards.

3. The globe is, more often than not, alone affected, but, not infrequently the globe and epididymis suffer together, and quite exceptionally the epididymis is attacked singly. The cord may rarely suffer, but the vas, vesicular seminales and prostate are not attacked.

4. The disease is very frequently, but not invariably, bilateral in its distribution, though one side may be more advanced in pathological changes than the other.

5. Hydrocele of the tunica vaginalis is not such an infrequent accompaniment of the malady as some writers would have us believe.

6. There is just a possibility that hydrocele of the cord may, in some instances, owe its origin to congenital syphilis.

7. The swelling of the testicle is a familiar one, it feels like scirrhous ; it may, or may not be nodular, the latter for the most part, and a fungus testis is sometimes seen.

8. The enlargement of the affected organ is usually not great and in fact there may be none at all.

9. In the large majority of instances the microscopical appearance is that of the simple inflammatory form, passing on to the development of fibrous tissue with consequent destruction of the gland, leading, possibly, if not attacked in time by suitable remedies, to impotence and sterility. The inflammatory form is akin to that observed in the liver. Gummata, on the other hand, are rare manifestations.

It is often very difficult to distinguish tuberculous disease of the testicles from the syphilitic malady.—*Archives Pediatrics.*

